# Whole genome sequencing of familial isolated oesophagus atresia uncover shared structural variants

**DOI:** 10.1186/s12920-020-00737-6

**Published:** 2020-06-26

**Authors:** Joakim Klar, Helene Engstrand-Lilja, Khurram Maqbool, Jonas Mattisson, Lars Feuk, Niklas Dahl

**Affiliations:** 1 Department of Immunology, Genetics and Pathology, Science for Life Laboratory, Uppsala, Sweden; 2 Department of Women’s and Children’s Health, Section of Pediatric Surgery, Uppsala University, SE-75185 Uppsala, Sweden

**Keywords:** Oesophagus atresia, Whole genome sequencing

## Abstract

**Background:**

Oesophageal atresia (OA) is a life-threatening developmental defect characterized by a lost continuity between the upper and lower oesophagus. The most common form is a distal connection between the trachea and the oesophagus, i.e. a tracheoesophageal fistula (TEF). The condition may be part of a syndrome or occurs as an isolated feature. The recurrence risk in affected families is increased compared to the population-based incidence suggesting contributing genetic factors.

**Methods:**

To gain insight into gene variants and genes associated with isolated OA we conducted whole genome sequencing on samples from three families with recurrent cases affected by congenital and isolated TEF.

**Results:**

We identified a combination of single nucleotide variants (SNVs), splice site variants (SSV) and structural variants (SV) annotated to altogether 100 coding genes in the six affected individuals.

**Conclusion:**

This study highlights rare SVs among candidate gene variants in our individuals with OA and provides a gene framework for further investigations of genetic factors behind this malformation.

## Background

Oesophageal atresia (OA) is the most common congenital anomaly of the oesophagus with an incidence of around 1 in 3500 births [1, 2]. The malformation is characterized by a distal tracheoesophageal fistula, classified as Gross type C, in 85% of all cases [3]. Isolated OA occurs in approximately 50% of cases whereas the remaining are syndromic [4–7]. Although the genetic basis for syndromic OA has been identified in a proportion of syndromic cases, the genetics behind isolated forms remains elusive. The recurrence risk for isolated OA is estimated to approximately 1% and twin studies have shown a concordance rate of 2.5% [8–10]. The few familial cases of isolated OA that are reported suggest an autosomal dominant inheritance with reduced penetrance [8, 11, 12] whereas epidemiological studies on isolated OA indicate a multifactorial aetiology, with a contribution from both gene variants and environmental factors [4]. A prior effort to unravel genetic factors behind isolated OA using SNP microarray analysis identified two distinct de novo CNVs in a cohort consisting of 129 cases [13]. When including syndromic OA, no consistent genomic region was identified. While the study provided important information, it is still unclear whether structural variants (SVs), small insertions/deletions (indels) and single nucleotide variants (SNVs), below detection limit of the DNA microarray used, are associated with the malformation.

Genetic variation in humans can be everything from rare to common, where variants usually associated with Mendelian traits tend to be rare whereas more frequent allele variants contribute risk for complex disease phenotypes [14]. The genetic architecture of both rare Mendelian diseases and multifactorial disorders such as birth defects may be explained by a continuum based primarily on the frequencies of the relevant variant alleles. Furthermore, incomplete penetrance for one or several common variants has inferred a shift beyond the classical concept of Mendelian inheritance [15, 16]. Complex traits can be seen as omnigenic and thereby driven by large numbers of variants of small effects and, in this context, we also need to identify modifier loci that contribute to penetrance of variation [17]. We therefore set out to analyse the genomic sequences of three families with recurrent cases of a low tracheoesophageal fistula (TEF), the most common form of congenital OA. In order to decipher gene variants in individuals with the malformation we performed whole genome sequencing of affected members and their parents. Bioinformatic analysis using a set of variant calling algorithms revealed a large number of candidate SNVs, indels, and SVs shared by affected and obligate carriers. Our data provide a selected set of candidate genetic variants to further identify, prioritize and test for genetic mechanisms associated with OA.

## Methods

### Patients

**Fig. 1:**
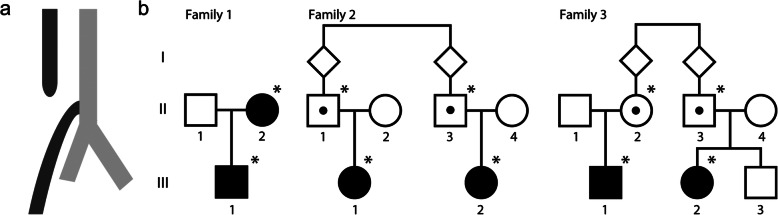
**Isolated oesophageal atresia gross type C segregating in three families**. **a** OA type C is illustrated by an upper and a lower oesophageal segment (dark grey) connected to the trachea (light grey) with a tracheoesophageal fistula. **b** Pedigrees of the three families included in the study. Affected individuals are indicated by filled squares (male) or circles (female). Assumed obligate carriers are indicated with a dot. *Sequenced individuals

We identified three families with recurrent and isolated OA treated in our tertiary paediatric surgical centre between 1994 and 2014 among a cohort of in total 55 cases with isolated OA Gross type C. Each of the three families comprised two individuals born with isolated OA. All the patients had an isolated OA with a lower tracheoesophageal fistula (Gross type C). None of them had other malformations or dysmorphic features, including VACTERL association or CHARGE syndrome (Fig. 1a).

In Family 1, the mother and her son were born with OA. In Family 2, two affected girls were second cousins and their fathers, respectively, were first cousins. In Family 3, a girl and a boy with OA were second cousins. The father of the girl and the mother of the boy were first cousins (Fig. 1b).

### Genetic analysis

Whole genome sequencing (WGS) was performed on peripheral blood DNA from the affected individuals (*n* = 6) and the presumed obligate and healthy carriers (*n* = 4). Sequencing libraries were prepared using the TruSeq PCRfree DNA sample preparation kit and sequenced using HiSeqX and paired-end 150 bp read length, v2.5 sequencing chemistry (Illumina). Quality control measurements are gathered using Qualimap v2.2. For data analysis we used Piper, a pipeline system developed and maintained at the National Genomics Infrastructure build on top of GATK Queue. For more information and the source code visit: www.github.com/NationalGenomicsInfrastructure/piper. Data has been aligned to the GRCh37.75 reference using BWA-MEM v0.7.12 [18] and we identified single nucleotide variants (SNVs) and indels using the Genome Analysis Toolkit (GATK v3.3). For deduplication PicardMarkDuplicates, available in Picard that is bundled with GATK (broadinstitute.github.io/picard). For the analysis of structural variants (SVs), we used Manta, Delly, CNVnator and Tiddit as described previously [19–23]. Manta and Delly calls SVs using combination of paired and split-reads and report breakpoints for SVs with strong evidence from split-reads whereas CNVnator uses read depth technique. TIDDIT is mainly designed to identify larger SVs (> 1 kb) using coverage and insert size distribution to identify SVs based on discordant read pairs. We collapsed SVs who had regions where both starts and ends were within 10 bp, as they likely represent the same called SV. All variants were annotated using ENSEMBL Variant Effect Predictor (VEP) [24]. Variants that potentially affect splicing were identified using SpliceAI (Score cut off 0.2 (high recall/likely pathogenic), 0.5 (recommended/pathogenic), and 0.8 (high precision/pathogenic)) [25]. We filtered SNVs and indels for frequency and sequencing errors using the Moon (www.diploid.com) software and against GnomAD [26]. The SVs were filtered against 1000 Genomes and the SweGen dataset using the aforementioned SV callers [23]. The SweGen dataset is the largest available cross-section of variation detected by the same WGS pipeline as we used in the Swedish population and can be seen as a population matched control dataset for our families. We annotated identified SVs with allele frequencies from 1000G, GnomAD and the SweGen dataset for all four SV callers.

Tissue expression of genes was investigated using the Genotype-Tissue Expression (GTEx; gtexportal.org) Project Portal of 05/08/19. We did pathway analysis using EnrichR (amp.pharm.mssm.edu/Enrichr). We investigated associations between candidate genes identified in our study and oesophageal disease using GeneDistiller [27]. A candidate gene list was generated in GeneDistiller by adding OMIM entries using the keyword ‘esophageal’ and manually adding genes previously associated with OA or similar phenotypes (complete reference gene list: *ALDH2, C2orf40, CHD7, COL4A6, CTAG1B, DEC1, DLEC1, EFTUD2, FANCB, FGF8, FOXC2, FOXF1, FOXL1, GAEC1, GER, GLI3, HOXD13, HSN1B, LZTS1, MTHFSD, MYCN, PTEN, RFX6, SOX2, SPG9, TMPRSS11A,* and *ZIC3*). Associations identified by GeneDistiller included Gene Reference into Function (GeneRIF) and Search Tool for the Retrieval of Interacting Genes/Proteins (STRING; string-db.org).

## Results

### Whole genome sequencing

Quality control measurements show that the mean coverage for the 10 samples was 32.6X (min 21.9X; max 39.9X) with a median insert size of 394 (min 371; max 419). The total number of reads was 1,057,991,431 (min 794,472,217; max 1,279,622,464) per sample and the number of aligned reads was 1,052,483,855 (min 791,300,824; max 1,270,865,080) corresponding to 99.5% aligned reads (min 99.3%; max 99.7%). The duplication rate was 35% (Standard deviation = 5.4%; min 28%; max 42%). We excluded sample bias by looking at the GC-content distribution compared to a pre-calculated distribution for the reference genome (hg19; Additional file 1).

### Single nucleotide variants (SNVs) and small insertions/deletions (indels)

**Fig. 2:**
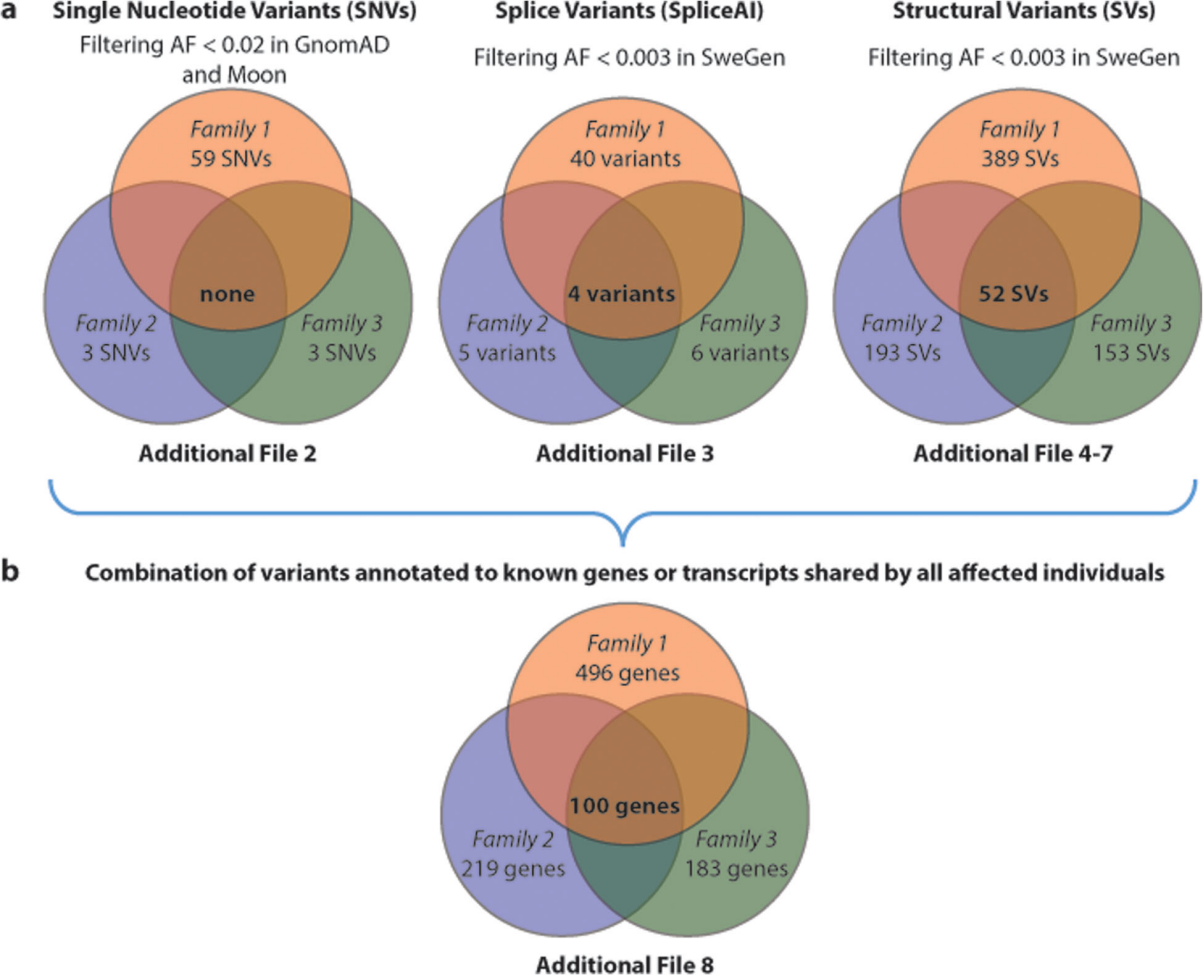
**Overview of variant calling in WGS data. a** Number and type of gene variants called in each of the three families segregating OA with number of shared variants. For SNVs and small indels, we identified 59 heterozygous variants in Family 1, 3 heterozygous variants in Family 2 and 3 heterozygous variants in Family 3 compatible with a dominant model. None of these variants were shared between all three families. For splice variants identified using SpliceAI, we identified 40 heterozygous variants in Family 1, 5 variants in Family 2 and 6 variants in Family 3. Four splice variants were shared by all three families. For structural variants we identified 389 SVs (339 DEL, 47 DUP and 3 INV) in Family 1, 193 SVs (172 DEL, 19 DUP and 2 INV) in Family 2 and 153 SVs (135 DEL, 17 DUP and 1 INV) in Family 3. In total, 52 SVs (43 DEL and 9 DUP) were shared by all three families. **b** Number of genes with annotated variants in each family and the number of genes shared by all affected individuals. When combining our data sets (**a**) we identified rare SNVs, splice variants or SVs that were annotated to in total 100 different genes in all three families. AF; allele frequency

After an initial filtering for uncommon variants (< 2% frequency in GnomAD) shared by the affected members of each family, we identified in total 345,156 (6604 coding) variants in Family 1, 199,780 (3450 coding) variants in Family 2 and 207,691 (3549 coding) SNVs variants in Family 3. We also performed a combined analysis using all three families and identified 109,712 (2098 coding) SNVs and indels shared by all six affected individuals. Since no shared homozygous, or compound heterozygous, variants were identified with an allele frequency less than 0.001 in GnomAD, we considered dominant acting variants and filtered for rare coding heterozygous variants present in all sequenced individuals. This approach revealed in total 47 heterozygous variants in coding sequences and splice sites. However, when filtering against the Moon database, which includes sequencing errors, all 47 variants showed an allele frequency of > 0.029. These high frequencies suggest that the variants are either benign or common sequencing errors. We therefore performed a re-analysis of each family separately. By applying all abovementioned filtering steps, we identified 59 variants in Family 1, three variants in Family 2 (in *KATNB1, ZMYND15* and *DHX33*) and three variants in Family 3 (in *BCAP29*, *DOCK4* and *PPP1R3A*) (Fig. 2a). All identified variants were heterozygous compatible with a dominant model (Additional file 2). We further analysed our data using SpliceAI in a similar fashion and identified 40 heterozygous splice-site variants in Family 1, 5 variants in Family 2 and 6 variants in Family 3. Four variants were shared by all 10 sequenced individuals (Fig. 2a). The variants have a predicted effect on splicing of the *PRIM2*, *FAM182B*, *MAP2K3* and *CCDC144NL* genes (Additional file 3). The shared variants identified in Family 2 and Family 3, respectively, were present also in the obligate carrier parents of affected cases. No variants identified was inherited from the non-sequenced parent, indicating co-segregation from the obligate carrier parents in family 2 and 3 and from the affected parent in family 1.

### Structural variants (SVs)

We then analysed our sequencing data for SVs. In total, we identified 5777 deletions (DEL), 1480 duplications (DUP) and 104 inversions (INV) that were detected in at least one sample. Filtering for SVs shared by all six affected individuals was performed as for SNVs and small indels (AF < 0.003 in SweGen which equals at most two individuals carrying the SV) resulting in a total of 52 SVs, i.e. 43 DEL and 9 DUP (Additional file 4). Analysis of SV shared by the two affected individuals in each separate family resulted in the identification of 339 DEL, 47 DUP and 3 INV in Family 1, 172 DEL, 19 DUP and 2 INV in Family 2 and 135 DEL, 17 DUP and 1 INV in Family 3 (Fig. 2a; Additional files 5, 6 and 7). All identified variants were found also in the parents of each family confirming segregation.

### Combination of variants in genes shared by all affected individuals

When combining our data sets we identified rare SNVs, splice variants or SVs that were annotated to in total 100 genes in all three families (Fig. 2b). The major proportion of variants consisted of SVs (involving 95 genes) followed by splicing (involving 4 genes). A single gene (*DOCK4*) showed a SNV (NM_014705.3:c.717G > C) in Family 3 in combination with a splice variant (acceptor gain) in Family 1 and a SV (intronic deletion of 33 bp) in Family 2 (Additional file 8). The 100 genes were then subject analysis by GeneDistiller to identify genes associated to the oesophagus. Two genes, *DEFB4A* and *PDS5B*, are associated (GeneRIF) with Esophageal squamous cell carcinoma (ESCC). A third gene, *PRKCZ*, is indirectly associated to the same disease in that it interacts (STRING) with the ESCC susceptibility gene *LZTS1.* The 100 shared genes are apparently diverse in function as we could not identify any significantly enriched category using EnrichR. Similarly, if we consider all genes from each family regardless whether they are shared (595 genes) we similarly did not find any significantly enriched category.

## Discussion

Epidemiological studies indicate that the causes of isolated OA are multifactorial similar to many other birth defects. We identified three families segregating isolated OA and performed whole genome sequencing on samples from family members in search for candidate gene variants with a relatively high penetrance for the malformation. The clinic was very similar between all three families described as OA Gross type C. However, all families was genetically analysed separately. Due to the fact that familial cases of OA is rare and that the patients came from the same population (Swedish), we also considered shared aetiology. A possible pattern of inheritance was autosomal dominant, with reduced penetrance in two families and, retrospectively, only genes with heterozygous variants were shared between individuals in each family. We hypothesized that shared rare variants in the two extended families 2 and 3, each one with affected second cousins, would bring stronger support for associated gene variants given the number of meiosis separating the cases. Due to the large number of variants identified in each of the small families (> 400 per family), we decided to focus the discussion on variants in genes shared by all three families. Bioinformatic analysis of WGS data disclosed 100 genes affected by a combination of SNVs, splice site variants and SVs in all six affected individuals (Fig. 2b). Our initial analysis of rare protein coding SNVs or small indel (filtered for an AF < 0.001) did not show any shared variant among all six cases. However, further analysis using SpliceAI revealed four heterozygous variants, none of them present in GnomAD, in the sequenced family members. The variants predict perturbed splicing of the *PRIM2*, *FAM182B*, *MAP2K3* and *CCDC144NL* genes, respectively. Notably, variants in *PRIM2* have been associated with a specific microbiome community of the oesophagus and *CCDC144NL* show differential expression in drug resistant oesophageal carcinoma cells (ESCC) [28, 29]. No connection could be found for the genes *FAM182B* and *MAP2K3* to oesophageal disease, although *MAP2K3* show a high level of expression in oesophageal tissue.

Importantly, the largest group of called variants that were shared among the six individuals with isolated OA and the four presumed carrier parents consists of SVs. Identification of SVs from short-read sequencing data is challenging as it yields a large amount of both false positive and false negative results. This is mainly due to repetitive elements that frequently result in ambiguities when trying to assign short read sequences to reference sequences. To this end, we combined four different callers, with their advantages and disadvantages, for the identification of SVs. This resulted in the identification of in total 52 shared SVs in all three families. Variants were identified in two gene regions with association to the oesophagus, namely *DEFB4A* and *PDS5B* previously associated with ESCC [30, 31] (Additional file 4). The *DEFB4A* gene (previously called *HBD2*) has an almost oesophagus specific expression and promotes both growth and invasion of oesophageal cancer [30, 32]. Moreover, allelic loss and altered expression of the *PDS5B* gene has been associated in ESCC [31]. A third gene, *PRKCZ* has a weak association to the oesophagus. This gene encodes for Protein kinase C zeta, a protein that interacts with Leucine zipper tumour suppressor 1 encoded by the gene *LZTS1* [33]. Somatic variants in *LZTS1* have been associated primary oesophageal cancer [34]. Interestingly, young adults with OA have a 100-fold increased prevalence of ESCC compared to the general population and a shared mechanism between the two disorders is plausible [35]. Cancer is usually associated with loss of function variants, while in our cases non-coding SVs may alter expression of genes that are required for proper specification and elongation of the oesophagus during embryogenesis, ultimately resulting in OA [36]. However, the high incidence of gastroesophageal reflux disease (GERD) in patients after OA repair may be an additional contributing factor to ESCC [37]. Recessive variants in the gene *TRAP1* have previously been associated with VACTERL, therefore it is possible that the heterozygous variant identified in this gene in Family 1 is a possible modifier of disease [38].

Our study adds to the few families reported with recurrent isolated OA and suggests contributing genetic factors behind the malformation. Furthermore, we applied a combination of bioinformatic tools on WGS data from affected families highlighting a large number of rare variants, primarily SVs, in individuals with the malformation in our cohort. Genome wide association studies (GWAS) of other non-syndromic congenital malformations such as cleft-lip-palate (CLP) and congenital heart disease have reported associations to numerous SNVs with significant effect sizes [39]. These risk variants occur frequently in the population and each one confers a modest effect size. In CLP, a GWAS study suggested a genomic region that is also vulnerable to rare variants [40]. However, risk loci identified by GWAS account for only a small fraction of the heritability of congenital malformations [41] and this is likely also for OA. While previous GWAS studies of different types of congenital malformations have provided important information on contributing genetic factors, they have not clarified the potential role of specific SVs in the aetiology of isolated birth defects and for some of the missing heritability [39]. Our study shows that the identification of rare SVs, whether in coding or non-coding regions, is possible using whole genome sequencing and novel bioinformatic pipe-lines.

## Conclusion

In summary, we explored the possible association between familial forms of isolated OA and rare gene variants using whole genome sequencing. In two out of the three families, the affected members were second cousins, whereas one family consisted of an affected mother and son. Among all variants (SNVs, splice sites and SVs) in 100 genes shared by the six individuals from the three families, we identified variants in three genes associated with oesophageal disease, namely *CCDC144NL, DEFB4A* and *PDS5B*, bringing further support for a molecular link between OA and oesophageal cancer. Furthermore, our study provides a data set for further computational and functional analyses of both isolated and syndromic OA. We anticipate that the identification of additional families segregating isolated OA, in combination with our findings, will facilitate the identification of specific gene variants contributing to this serious malformation.

## Supplementary information

**Additional file 1 Quality control of GC-content distribution.** The distribution of GC content of mapped reads for the samples (orange) indicate expected distribution compared to a pre-calculated GC distribution for the reference genome (hg19; blue). The bars indicate standard deviation (SD) for the samples (*n* = 10).

**Additional file 2 Variants identified using a dominant model.** All rare variants (< 2% frequency in GnomAD) identified following a dominant segregation pattern in the families.

**Additional file 3 SpliceAI variants identified using a dominant model.** All rare predicted splice variants (< 2% frequency in GnomAD) identified following a dominant segregation pattern in the families.

**Additional file 4 SVs identified.** All rare SVs (AF < 0.003 in SweGen) shared by the affected individuals in the families.

**Additional file 5 SVs identified in Family 1.** All rare SVs (AF < 0.003 in SweGen) shared by the affected individuals in Family 1.

**Additional file 6 SVs identified in Family 2.** All rare SVs (AF < 0.003 in SweGen) shared by the affected individuals in Family 2.

**Additional file 7 SVs identified in Family 3.** All rare SVs (AF < 0.003 in SweGen) shared by the affected individuals in Family 3.

**Additional file 8 Summary of the type of variants in shared genes between all families.** Rare SNVs, splice variants or SVs that were annotated to in total 100 genes in all three families.

## Data Availability

The datasets during and/or analysed during the current study available from the corresponding author on reasonable request. The datasets generated during the current study submitted to The European Genome-phenome Archive (EGA; ID EGAS00001004394). GRCh37.75 reference is available from ENSEMBL (ftp://ftp.ensembl.org/pub/release-75). The SweGen Variant Frequency Dataset is available to the scientific community through the website swefreq.nbis.se and the National Bioinformatics Infrastructure Sweden (NBIS) DOI repository (doi:10.17044/NBIS/G000003) upon registration and agreement to terms and conditions for data download. Allele frequencies from GnomAD and 1000G was retrieved using the ENSEMBL Variant Effect Predictor (VEP). The GnomAD data are available from gnomad.broadinstitute.org (direct link: SNV frequency file from storage.googleapis.com/gnomad-public/release/2.1.1/vcf/genomes/gnomad.genomes.r2.1.1.sites.vcf.bgz and SV frequency file from storage.googleapis.com/gnomad-public/papers/2019-sv/gnomad_v2.1_sv.sites.vcf.gz). The 1000G data is available from internationalgenome.org/data (direct link: frequency file from ftp://ftp.ensembl.org/pub/grch37/current/variation/gvf/homo_sapiens/1000GENOMES-phase_3.gvf.gz).

## Abbreviations

OA: Oesophageal atresia
TEF: Tracheoesophageal fistula
SNVs: Single nucleotide variants
SSV: Splice site variants
SV: Structural variants
indels: Small insertions/deletions
WGS: Whole genome sequencing
DEL: Deletions
DUP: Duplications
INV: Inversions
ESCC: Esophageal squamous cell carcinoma
STRING: Search Tool for the Retrieval of Interacting Genes
gnomAD: The Genome Aggregation Database
GERD: Gastroesophageal reflux disease
GWAS: Genome wide association studies
CLP: Cleft-lip-palate
