# Supplementary material for: Whole genome sequencing of familial isolated oesophagus atresia uncover shared structural variants
Source: BMC Med Genomics. 2020 Jun 26;13:85. doi: 10.1186/s12920-020-00737-6 (PMC7318369; doi:10.1186/s12920-020-00737-6)
Supplement: Supplementary file 1 — Additional file 1 Quality control of GC-content distribution. The distribution of GC content of mapped reads for the samples (orange) indicate expected distribution compared to a pre-calculated GC distribution for the reference genome (hg19; blue). The bars indicate standard deviation (SD) for the samples (n = 10). [file 12920_2020_737_MOESM1_ESM.pdf]

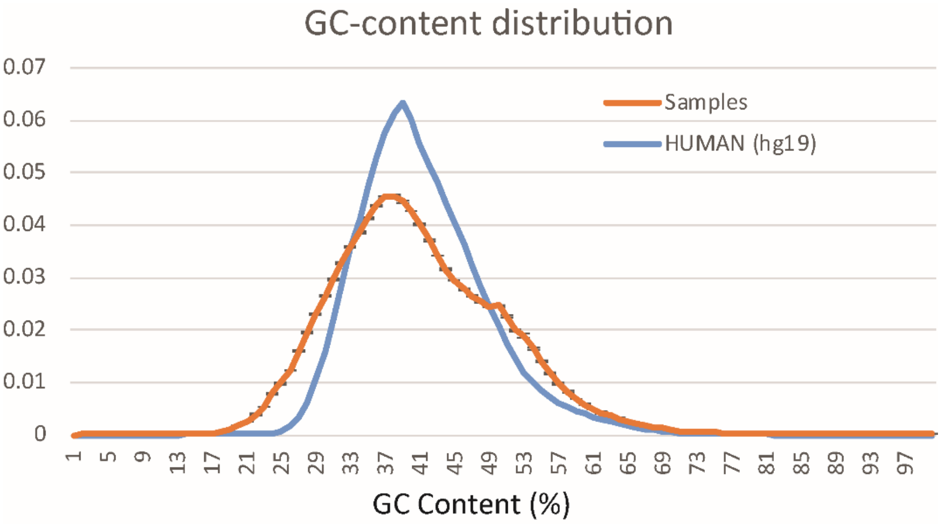

Additional File 1: Quality control of GC-content distribution. The distribution of GC content of mapped reads for the samples (orange) indicate expected distribution compared to a pre-calculated GC distribution for the reference genome (hg19; blue). The bars indicate standard deviation (SD) for the samples (n=10).
